# Supplementary material for: Evolution of Neo-RAS-WT in Circulating Tumor DNA from First-Line to Subsequent Therapies in Metastatic Colorectal Cancer
Source: Cancers (Basel). 2025 Mar 22;17(7):1070. doi: 10.3390/cancers17071070 (PMC11987899; doi:10.3390/cancers17071070)
Supplement: Supplementary file 1 [file cancers-17-01070-s001.zip › cancers-3476730-supplementary.pdf]

Table S1. RAS mutation types and RAS mutant allele fraction detected on primary tumor tissues

pts	tissue mutation	MAF	first-line baseline ctDNA	PD first-line	second-line baseline ctDNA	PD second-line	third-line baseline ctDNA	PD third-line
1	*NRAS G12D*	0,25%	*NRAS G12D*	*RAS WT*	*RAS WT-PIK3CA*	*RAS WT*	*NRAS Q61L*	*NRAS Q61L*
2	*NRAS A146T*	0,10%	*NRAS A146T*	*RAS WT*	*NRAS A146T*	*NRAS A146T*	*NRAS A146T*	*NRAS A146T*
3	*KRAS Q61H*	22.5%	*KRAS Q61H*	*RAS WT*	*RAS WT-IDH1-FGFR3*	*RAS WT*	*KRAS Q61R/L*	*KRAS Q61R/L*
4	*KRAS A146T*	0,13%	*KRAS A146T*	*RAS WT*	*RAS WT-PIK3CA*	*RAS WT*	*KRAS A146T*	*KRAS A146T*
5	*KRAS G13D*	36.3%	*KRAS G13D*	*KRAS G13D*	*KRAS G13D*	*KRAS G13D*	*KRAS G13D*	*RAS WT*
6	*KRAS G12S*	17.2%	*KRAS G12S*	*KRAS G12S*	*KRAS G12S*	*KRAS G12S*	*KRAS G12S*	*KRAS G12S*
7	*KRAS G12V*	29.7%	*KRAS G12V*	*KRAS G12V*	*KRAS G12V*	*KRAS G12V*	*RAS WT*	*RAS WT*
8	*KRAS G13D*	31.2%	*KRAS G13D*	*KRAS G13D*	*KRAS G13D*	*KRAS G13D*	*KRAS G13D*	*KRAS G13D*
9	*KRAS G12V*	13.9%	*KRAS G12V*	*KRAS G12V*	*KRAS G12V*	*RAS WT*	*KRAS G12V*	*KRAS G12V*
10	*KRAS G12R*	0,40%	*KRAS G12R*	*KRAS G12R*	*KRAS G12R*	*KRAS G12R*	*KRAS G12R*	*RAS WT*
11	*KRAS G12D*	33.5%	*KRAS G12D*	*KRAS G12D*	*KRAS G12D*	*RAS WT*	*RAS WT-MET*	*KRAS G12D*
12	*KRAS G12D*	10.7%	*KRAS G12D*	*KRAS G12D*	*KRAS G12D*	*KRAS G12D*	*KRAS G12D*	*KRAS G12D*
13	*KRAS G12D*	0,38%	*KRAS G12D*	*KRAS G12D*	*KRAS G12D*	*RAS WT*	*RAS WT-MET*	*KRAS G12D*
14	*KRAS G12D*	29.8%	*KRAS G12D*	*KRAS G12D*	*KRAS G12D*	*KRAS G12D*	*KRAS G12D*	*KRAS G12D*
15	*KRAS G12A*	40.6%	*KRAS G12A*	*KRAS G12A*	*KRAS G12A*	*KRAS G12D*	*KRAS G12D*	*KRAS G12D*
16	*KRAS Q61H*	0,12%	*KRAS Q61H*	*NRAS A146T*	*NRAS A146T*	*NRASA146T*	*NRAS A146T*	*NRAS A146T*
17	*KRAS K117N*	0,20%	*KRAS K117N*	*RAS WT*	*RAS WT-PIK3CA*	*KRAS G12D*	*KRAS G12D*	*KRAS G12D*
18	*KRAS A146V*	25.8%	*KRAS A146V*	*KRAS G12D*	*KRAS G12D*	*KRAS G12D*	*KRAS G12D*	*KRAS G12D*
19	*KRAS G12A*	22.9%	*KRAS G12A*	*BRAF V600E*	*BRAF V600E*	*BRAF V600E*	*BRAF V600E*	*BRAF V600E*
20	*KRAS G12D*	8.5%	*KRAS G12D*	*KRAS G12D*	*KRAS G12D*	*KRAS G12D*	*KRAS G12D*	*KRAS G12D*
21	*KRAS G12C*	11.7%	*KRAS G12C*	*KRAS G12C*	*KRAS G12C*	*RAS WT*	*RAS WT*	*KRAS G12C*
22	*KRAS G12V*	0,42%	*KRAS G12V*	*KRAS G12V*	*KRAS G12V*	*NRAS A146T*	*NRAS A146T*	*NRAS A146T*
23	*KRAS G12C*	34.2%	*KRAS G12C*	*KRAS G12C*	*KRAS G12C*	*KRAS G12C*	*KRAS G12C*	*KRAS G12C*
24	*KRAS G12C*	0,28%	*KRAS G12C*	*KRAS Q61H*	*KRAS Q61H*	*KRAS Q61H*	*KRAS Q61H*	*RAS WT*
25	*KRAS G12C*	31.7%	*KRAS G12C*	*KRAS G12C*	*KRAS G12C*	*RAS WT*	*RAS WT*	*RAS WT*
26	*KRAS A146T*	39.4%	*KRAS A146T*	*KRAS A146T*	*KRAS A146T*	*KRAS A146T*	*KRAS A146T*	*KRAS A146T*
27	*KRAS G12A*	0,06%	*KRAS G12A*	*KRAS G12A*	*KRAS G12A*	*RAS WT*	*KRAS G12A*	*KRAS G12A*
28	*KRAS G12V*	18.2%	*KRAS G12V*	*KRAS G12V*	*KRAS G12V*	*KRAS G12V*	*KRAS G12V*	*KRAS G12V*
29	*KRAS G12D*	17.5%	*KRAS G12D*	*KRAS G12D*	*KRAS G12D*	*KRAS G12D*	*KRAS G12D*	*KRAS G12D*
30	*BRAF V600E*	31.6%	*BRAF V600E*	*RAS WT*	*RAS WT- PIK3CA*	*RAS WT*	*BRAF V600E*	*BRAF V600E*
31	*NRAS A146T*	0,25%	*NRAS A146T*	*NRAS A146T*	*NRAS A146T*	*RAS WT*	*NRAS A146T*	*NRAS A146T*
32	*KRAS G12D*	31.5%	*KRAS G12D*	*KRAS G12D*	*KRAS G12D*	*KRAS G12D*	*KRAS G12D*	*KRAS G12D*
33	*KRAS A146V*	24.5%	*KRAS A146V*	*KRAS A146V*	*KRAS G12V*	*KRAS G12V*	*KRAS G12V*	*KRAS G12V*
34	*KRAS G12D*	0,19%	*KRAS G12D*	*KRAS G12D*	*KRAS G12D*	*KRAS G12D*	*KRAS G12D*	*KRAS G12D*
35	*KRAS G12D*	0,40%	*KRAS G12D*	*KRAS G12D*	*KRAS G12D*	*KRAS G12D*	*KRAS G12D*	*KRAS G12D*
